# Mass Spectrometry and Machine Learning Reveal Determinants of Client Recognition by Antiamyloid Chaperones

**DOI:** 10.1016/j.mcpro.2022.100413

**Published:** 2022-09-15

**Authors:** Nicklas Österlund, Thibault Vosselman, Axel Leppert, Astrid Gräslund, Hans Jörnvall, Leopold L. Ilag, Erik G. Marklund, Arne Elofsson, Jan Johansson, Cagla Sahin, Michael Landreh

**Affiliations:** 1 Department of Biochemistry and Biophysics, Stockholm University, Stockholm, Sweden; 2 Department of Microbiology, Tumor and Cell Biology, Karolinska Institutet, Stockholm, Sweden; 3 Department of Medical Biochemistry and Biophysics, Karolinska Institutet, Stockholm, Sweden; 4 Department of Materials and Environmental Chemistry, Stockholm University, Stockholm, Sweden; 5 Department of Chemistry - BMC, Uppsala University, Uppsala, Sweden; 6 Science for Life Laboratory and Department of Biochemistry and Biophysics, Stockholm University, Solna, Sweden; 7 Department of Biosciences and Nutrition, Karolinska Institutet, Neo, Huddinge, Sweden; 8 Department of Biology, University of Copenhagen, Denmark

**Keywords:** structural proteomics, machine learning, protein misfolding, molecular chaperones, Aβ, amyloid β, AD, Alzheimer’s disease, AF2, AlphaFold2, βLG, β-lactoglobulin, CCS, cross-section, CTC, C-terminal region of proSP-C, ESI, electrospray ionization, HDX, hydrogen–deuterium exchange, IM, ion mobility, MS, mass spectrometry, proSP-C, proform of lung surfactant protein C, TTR, transthyretin

## Abstract

The assembly of proteins and peptides into amyloid fibrils is causally linked to serious disorders such as Alzheimer’s disease. Multiple proteins have been shown to prevent amyloid formation *in vitro* and *in vivo*, ranging from highly specific chaperone–client pairs to completely nonspecific binding of aggregation-prone peptides. The underlying interactions remain elusive. Here, we turn to the machine learning–based structure prediction algorithm AlphaFold2 to obtain models for the nonspecific interactions of β-lactoglobulin, transthyretin, or thioredoxin 80 with the model amyloid peptide amyloid β and the highly specific complex between the BRICHOS chaperone domain of C-terminal region of lung surfactant protein C and its polyvaline target. Using a combination of native mass spectrometry (MS) and ion mobility MS, we show that nonspecific chaperoning is driven predominantly by hydrophobic interactions of amyloid β with hydrophobic surfaces in β-lactoglobulin, transthyretin, and thioredoxin 80, and in part regulated by oligomer stability. For C-terminal region of lung surfactant protein C, native MS and hydrogen–deuterium exchange MS reveal that a disordered region recognizes the polyvaline target by forming a complementary β-strand. Hence, we show that AlphaFold2 and MS can yield atomistic models of hard-to-capture protein interactions that reveal different chaperoning mechanisms based on separate ligand properties and may provide possible clues for specific therapeutic intervention.

## Highlights

• Chaperone–client interactions are hard to capture in structural biology.
• Machine learning can generate possible chaperone complex structures.
• Mass spectrometry can identify complex architectures and interaction sites.
• The combination reveals how specific and nonspecific chaperone interactions differ.

## Graphical Abstract

**Figure:**
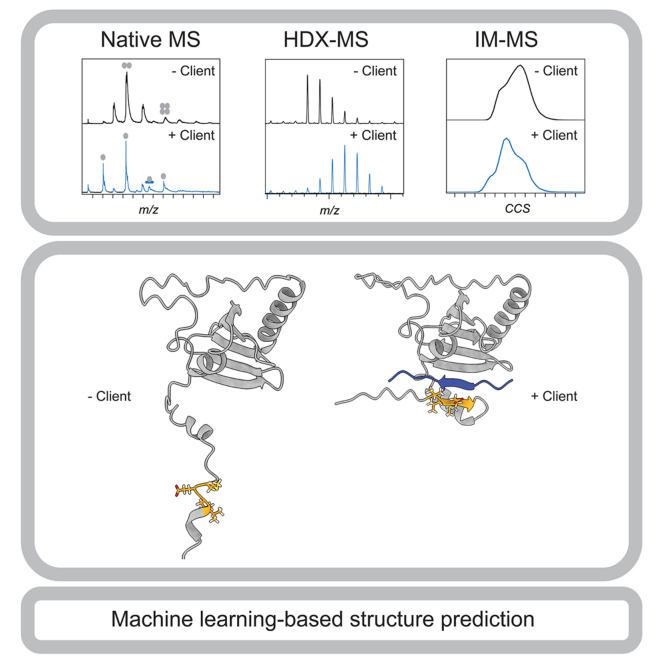

## In Brief

A wide range of proteins can prevent amyloid formation *in vitro* and *in vivo*, but the exact nature of these interactions is unclear. By combining machine learning–based structure prediction of protein complexes with mass spectrometry, we can discern the molecular determinants of specific and nonspecific chaperone–client interactions.

Fibrillar protein aggregates have been observed in a wide range of diseases, including cancer, systemic amyloidosis, and interstitial lung disease (1, 2, 3). They are, however, mostly associated with neurodegenerative disorders, such as Alzheimer’s disease (AD) and Parkinson’s disease (4). The assembly of proteins or peptides into highly ordered β-sheet-rich amyloid fibrils can give rise to pathological conditions through the formation of toxic intermediates that cause cell damage, loss of function of the aggregating protein, and/or accumulation of fibrillar material (5, 6). Consequently, development of therapeutic strategies has often focused on preventing or reversing the aggregation process (7). Some clues for targeted intervention can be gathered from nature, as the ability to form fibrils is widespread in the proteome (8). Aggregation processes are however under normal cellular conditions tightly regulated to prevent detrimental effects. Several proteins have been found to have antiamyloid activity by blocking aggregation or stabilizing the native state of their client. Protein systems with antiamyloid activity are diverse, ranging from dedicated chaperones such as small heat shock proteins to proteins with unrelated physiological functions, like serum albumin (9, 10, 11). However, amyloid-forming proteins have a challenging chemical nature, often combining conformational flexibility and poor solubility. Therefore, detailed structural information that exactly reveals how proteins counteract fibril formation has remained scarce.

We have previously reported antiamyloid activity in proteins whose biological contexts suggest significant differences in client specificity. For example, we have shown that the bovine whey protein β-lactoglobulin (βLG), along with several other analytical protein standards, such as bovine serum albumin, lysozyme, and pyruvate kinase, can prevent aggregation of the model amyloid β (Aβ) peptide *in vitro* (10). NMR spectroscopy analysis revealed that monomeric Aβ broadly interacts with these proteins *via* hydrophobic interactions. The chaperone effect thus arises from indiscriminate contact and is most pronounced at or above near-stoichiometric ratios between chaperone and client. Several nonchaperone proteins with antiamyloid activity have been implicated in protein aggregation diseases like AD. A prominent example is transthyretin (TTR), a transport protein that is itself related to amyloid disease upon destabilization of its native tetrameric state. TTR is known to inhibit *in vitro* aggregation of other amyloidogenic species including Aβ (12, 13), even though the exact interaction mode is disputed (14, 15, 16). Another example is T80, a thioredoxin variant that can prevent Aβ aggregation *in vitro*, and the levels of which are decreased in the cerebral spinal fluid of AD patients (17). T80 is produced by α-secretase cleavage of the C-terminal α-helix of thioredoxin, exposing a hydrophobic patch that has been linked to its antiamyloid activity (17).

While βLG, TTR, and T80 are examples of nonspecific antiamyloid chaperones, the proform of lung surfactant protein C (proSP-C) is the exact opposite. SP-C, a highly aggregation-prone transmembrane peptide located in the lipid bilayers of the alveolae, is synthesized with a C-terminal BRICHOS domain, homologs of which have been identified in several proteins associated with neurodegeneration and cancer (18). ProSP-C BRICHOS binds to the polyvaline region of SP-C, preventing its assembly into fibrils (19, 20). Mutations in the C-terminal region of proSP-C (CTC), composed of BRICHOS and a disordered region, give rise to SP-C amyloid deposits and lead to interstitial lung disease (21). X-ray crystallography revealed that proSP-C BRICHOS, containing a central β-sheet flanked by two helices, assembles into trimers (21). SP-C with its BRICHOS domain thus represents an example of a highly specific partner in a chaperone–client pair (22). The proSP-C BRICHOS domain is however also known to inhibit fibrilization of other amyloid-forming peptides, such as the Aβ peptide, indicating that the chaperone is capable of recognizing common amyloidogenic motifs (22). Although the differences in chaperone specificity between βLG and CTC are obvious from their biological contexts, no detailed models have been available that could reveal the structural determinants of their specificity, as data from NMR and X-ray crystallography have proven insufficient to solve the structures of both complexes.

Recently, the arrival of machine learning–driven protein structure prediction algorithms such as AlphaFold2 (AF2) and RosettaFold has enabled modeling of previously inaccessible protein complexes with an accuracy that rivals experimentally determined structures (23, 24). Thus, AF2 has also been found to reliably dock short peptides into binding pockets of folded protein domains (25). However, predictions are based on user input and will suggest the most likely structure of any protein–peptide pair provided. It can deliver seemingly plausible models for protein interactions not occurring under native conditions, and the resulting models require therefore further experimental validation. Mass spectrometry (MS) offers several good analytical strategies complementary to AF2 (26, 27):

1. The use of physiological buffer conditions and carefully tuned instrument parameters have enabled us to preserve noncovalent interactions during MS analysis. The mass of intact complexes thus reveals protein and ligand interactions. The approach commonly termed “native MS” can be employed to determine binding stoichiometries and relative complex stabilities.
2. In ion mobility (IM) measurements, protein ions are separated according to their collision cross-sections (CCSs) in a gas-filled cell, which provides information about the spatial arrangement of their components. This method can reveal conformational changes and topologies upon ligand binding in protein complexes.
3. Labeling in solution, when hydrogens in the protein backbone are exchanged for deuterium (HDX), can be paired with MS to reveal the spatial distribution of the labels. HDX-MS thus gives information about conformational dynamics of interaction sites.

Here, we have used a combination of AF2 (with its models of protein complexes) and structural MS (with its information on stoichiometries, topologies, and interaction sites) to unravel differences in antiamyloid mechanisms. Using βLG, TTR, and Aβ, as well as CTC and polyvaline, we show how client β-strand propensity and hydrophobicity drive nonspecific and specific chaperone interactions, respectively.

## Experimental Procedures

### Protein Preparations

βLG was purchased from Sigma, and wildtype recombinant Aβ(1–40) as a lyophilized powder was purchased from Alexo-Tech AB. Scrambled recombinant Aβ, with the sequence KVKGLIDGAHIGDLVYEFMDSNSFR EGVGAGHVHVAQVEF, was purchased from rPeptide as a lyophilized powder. The peptides were dissolved in 6 M guanidine hydrochloride, purified by size-exclusion chromatography on a Superdex 75 Increase 10/300 GL column in 200 mM ammonium acetate (pH 6.9) to remove aggregated material, and stored on ice until analysis. Recombinant human T80 was purchased form R&D Systems, and recombinant human TTR from Alexo-Tech AB. CTC was prepared as described previously (21). Amidated and acetylated KKVVVVVVVKK (V7) and KKAAAAAAAKK (A7) were purchased from Thermo Electron.

### Native MS

Native IM-MS of βLG and TTR was performed using a Waters Synapt G2S traveling-wave IM mass spectrometer, and native MS of T80 was performed on a Waters Synapt G1 traveling-wave IM mass spectrometer. Samples were ionized using offline borosilicate emitters (Thermo). The capillary voltage was 1.2 to 1.5 kV, and the cone voltage was 20 V. The source temperature was 25 °C, and the source pressure was 3.4 mbar. Wave height and wave velocity were 35 V and 700 m/s in the IM spectrometry cell and 10 V and 248 m/s in the transfer cell. The IM spectrometry gas was nitrogen with a flow of 50 ml/min, and the trap gas was argon with a flow of 10 ml/min. Mass spectra were processed using MassLynx 4.1 (Waters). IM-MS data were analyzed using PULSAR (version 2.0, 2018) (28). Drift tube CCS values for βLG and TTR were used to calibrate T-wave data. Theoretical CCS values were computed using projection approximation in IMPACT (29) and scaled with the empirical factor of 1.14 (30). Native MS of CTC was performed on a Q-ToF Ultima API mass spectrometer (Waters) equipped with a Z-spray source using offline borosilicate emitters (Thermo). The source temperature was 30 °C, the capillary voltage was 1.5 kV, and the cone voltage was 20 V, respectively. The source pressure was maintained at 7 mbar. The mass spectrometer was operated in single-reflector mode.

### HDX-MS

For HDX-MS of CTC, a CTC stock solution with a concentration of 0.9 mM (determined by absorption at 280 nm) was diluted in deuterated Tris buffer to a final deuterium content of 92.5% and protein concentration of 30 μM. For peptide interaction studies, V7 or A7 was preincubated with CTC for 10 min at 22 °C at a molar ratio of 1:1 prior to deuteration. Aliquots of 20 μl were collected from separate incubations after 1, 5, 10, 30, and 60 min. Fully deuterated protein was prepared by freeze-drying of a CTC aliquot followed by resuspension in 99.9% deuterium oxide and incubation for 4 h at 50 °C. Deuterium exchange was quenched by transfer of aliquots to chilled Eppendorf tubes containing 0.5 μl of 5% TFA (Merck), subsequent vortexing, and freezing in liquid nitrogen until analysis. For MS analysis, deuterated aliquots were thawed and injected into an HPLC system submersed in an ice bath. Proteins were digested online in a Porozyme Immobilized Pepsin Cartridge (Applied Biosystems) operated at 17 μl/min in 0.05% TFA. The peptides obtained were desalted for 10 min using a Waters Symmetry C18 trap column and eluted in a single step with 70% acetonitrile in 0.1% formic acid at a flow rate of 17 μl/min. Samples were delivered to the mass spectrometer through a 50 μm tapered tip emitter (New Objective), and spectra were acquired on a Waters Ultima API mass spectrometer (Waters) equipped with a Z-spray source at a source temperature of 80 °C, a capillary voltage of 1.7 kV, and cone and RF lens 1 potentials of 100 and 38 V, respectively. The mass scale was calibrated using [Glu1]-fibrinopeptide B. Scans were acquired for 5 min at a rate of one scan per 2 s between 300 and 2000 *m/z*.

### AlphaFold2 Predictions

AF2 predictions were generated using ColabFold (https://colab.research.google.com/github/sokrypton/ColabFold/blob/main/AlphaFold2.ipynb), version 1.4, with default settings (five models, no AMBER step). Structures were visualized with ChimeraX, version 1.3 (https://www.cgl.ucsf.edu/chimerax).

### Experimental Design and Statistical Rationale

Native MS and IM-MS experiments were repeated between two and four times, and no significant deviations were observed when identical instrument parameters were used. HDX-MS datasets were recorded in triplicates (three separate deuterium incubations). Deuteration was calculated as the average and standard deviation of the *m/z* values of the isotope envelope centroids on experimental triplicates using the Waters MassLynx software package. Data were plotted using the Prism software (GraphPad Software, Inc) as average *m/z* and standard deviation from three independent repeats. Deuteration curves were fitted for one-phase association (with near-maximum deuteration reached at the first time point) or two-phase association (with an observable time-dependent increase).

## Results and Discussion

### The Nonchaperone Protein βLG Inhibits Aβ Aggregation *Via* Binding to Hydrophobic Surfaces

**Fig. 1:**
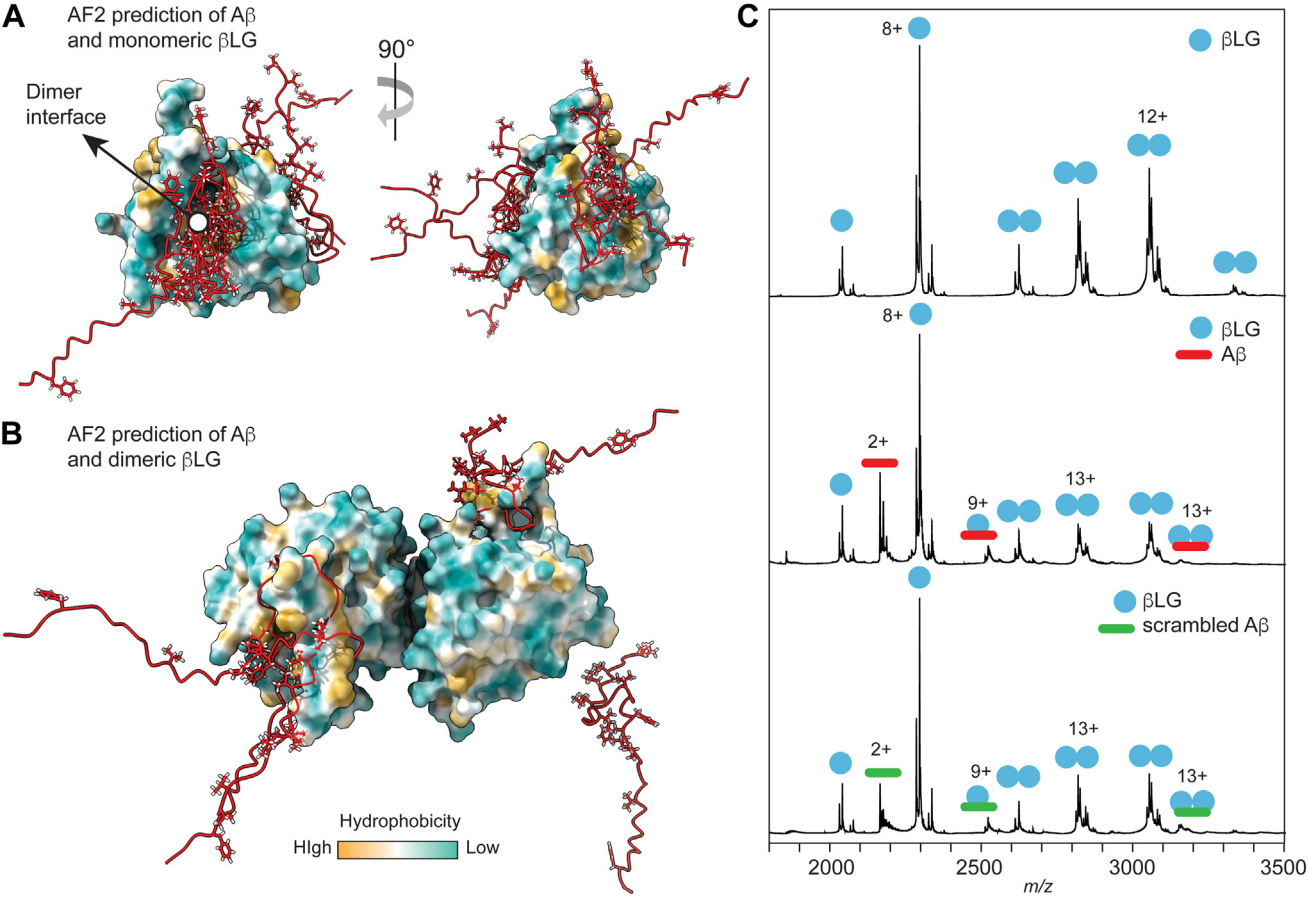
**Interactions between Aβ and βLG.***A*, the overlay of the top five AF2 models of monomeric Aβ (shown in *red* with hydrophobic residues rendered as *sticks*) bound to βLG (rendered as hydrophobicity surfaces) show orientation of hydrophobic residues toward hydrophobic surfaces including the exposed dimer interface. *B*, the top four AF2 predictions of dimeric βLG with Aβ show random placement of Aβ on hydrophobic patches of the dimer surface. Coloring as in (*A*). *C*, native MS analysis shows pronounced binding of Aβ to βLG monomers and a reduced amount of dimers. Replacement of Aβ with a scrambled Aβ sequence also results in complex formation. β, amyloid β; AF2, AlphaFold2; βLG, β-lactoglobulin; MS, mass spectrometry.

The ability of βLG to prevent Aβ aggregation has previously been attributed to nonspecific interactions that involve all residues of the Aβ peptide (10). To now obtain a more detailed insight into this association, we predicted the structure of βLG monomers and dimers in the presence and absence of Aβ (Fig. 1*A*). The AF2 model of dimeric βLG is in near-perfect agreement with the crystal structure giving an RMSD of 0.674 Å excluding the disordered N-terminal region. We then predicted the structures of monomeric and dimeric βLG with Aβ. Overlaying the five AF2 models of the 1:1 βLG–Aβ heterocomplex suggest Aβ binding mostly to the βLG dimer interface of βLG and to a lesser extent to other hydrophobic regions of the protein (Fig. 1*A*). Similarly, the predicted complex of dimeric βLG with monomeric Aβ did not indicate a preferred location for the peptide but rather a random placement with high positional alignment error scores, in line with the nonspecific association (Figs. 1*B* and S1). A surprising observation was that Aβ appeared to distort the prediction of the dimer in some cases, although these models have high positional alignment error scores even for the dimer interface (supplemental Fig. S1). To test these models, we turned to native MS. Mass spectra recorded in the presence of Aβ at physiological pH showed a decrease in the intensity of the βLG dimer peaks. We detected peaks corresponding in mass to a complex of Aβ with dimeric as well as with monomeric βLG, which were further confirmed by MS/MS (Figs. 1*C* and S1). Aβ with a scrambled sequence (Aβ_Scr_) gave the same binding pattern as wildtype Aβ (Fig. 1*C*). This observation supports the idea that the interaction is not sequence specific, as Aβ and Aβ_Scr_ have the exact same average hydrophobicity but different sequences. AF2 indeed suggests very similar interactions between Aβ_Scr_ and βLG to what was observed with the native Aβ peptide (supplemental Fig. S2). Binding does in both cases involve the most hydrophobic segments of the peptides, with Aβ_Scr_ having a more even distribution of hydrophobicity compared with Aβ (supplemental Fig. S2).

As the next step, we used IM-MS to determine the CCSs of the βLG–Aβ complexes and compared them with the data from the AF2 predictions. The experimental CCS of 2382 Å^2^ for the 9+ charge state of the 1:1 complex agrees reasonably well with the calculated CCS of 2290 Å^2^ for the top-scoring AF2 prediction. For Aβ bound to the βLG dimer, we obtained a CCS of 3716 Å^2^ for the 13+ ion, again similar to the expected CCS of 3850 Å^2^ for the highest-scoring complex model. Despite this agreement, it must be kept in mind that CCS data are not specific to each complex architecture. Even structurally dissimilar complexes can have near-identical CCSs. This point becomes clear when comparing the top-scoring models for Aβ bound to βLG, which have the flexible N-terminal segment of Aβ, collapsed on the surface of βLG. Such a collapse is likely to occur in the gas phase for all the proposed complex structures (31), meaning any of the proposed complexes will likely have similar CCS values regardless of the placement of the Aβ peptide. In summary, the insights from AF2, native MS, and IM-MS show that βLG captures Aβ monomers through nonspecific interactions that are likely to involve the exposed dimer interface.

It should be noted that nonspecific protein–ligand interactions are a well-known phenomenon in native MS (*e.g.*, (32)). Nonspecific interactions in solution and during electrospray ionization (ESI) are broadly similar, as both are driven by complementary charges, hydrogen bonds, and hydrophobic interactions between ligand and protein. The ESI process can give rise to nonspecific adducts if protein and ligand are present in the same electrospray droplet. The likelihood of this scenario has been estimated to increase at ligand and protein concentrations above 50 μM (33). Although the concentrations used in this study are lower (supplemental Table S1), we cannot exclude that complex formation between Aβ and βLG occurs at least in part during ESI.

### Binding of Monomeric Aβ to TTR and T80 Requires Exposed Subunit Interfaces

**Fig. 2:**
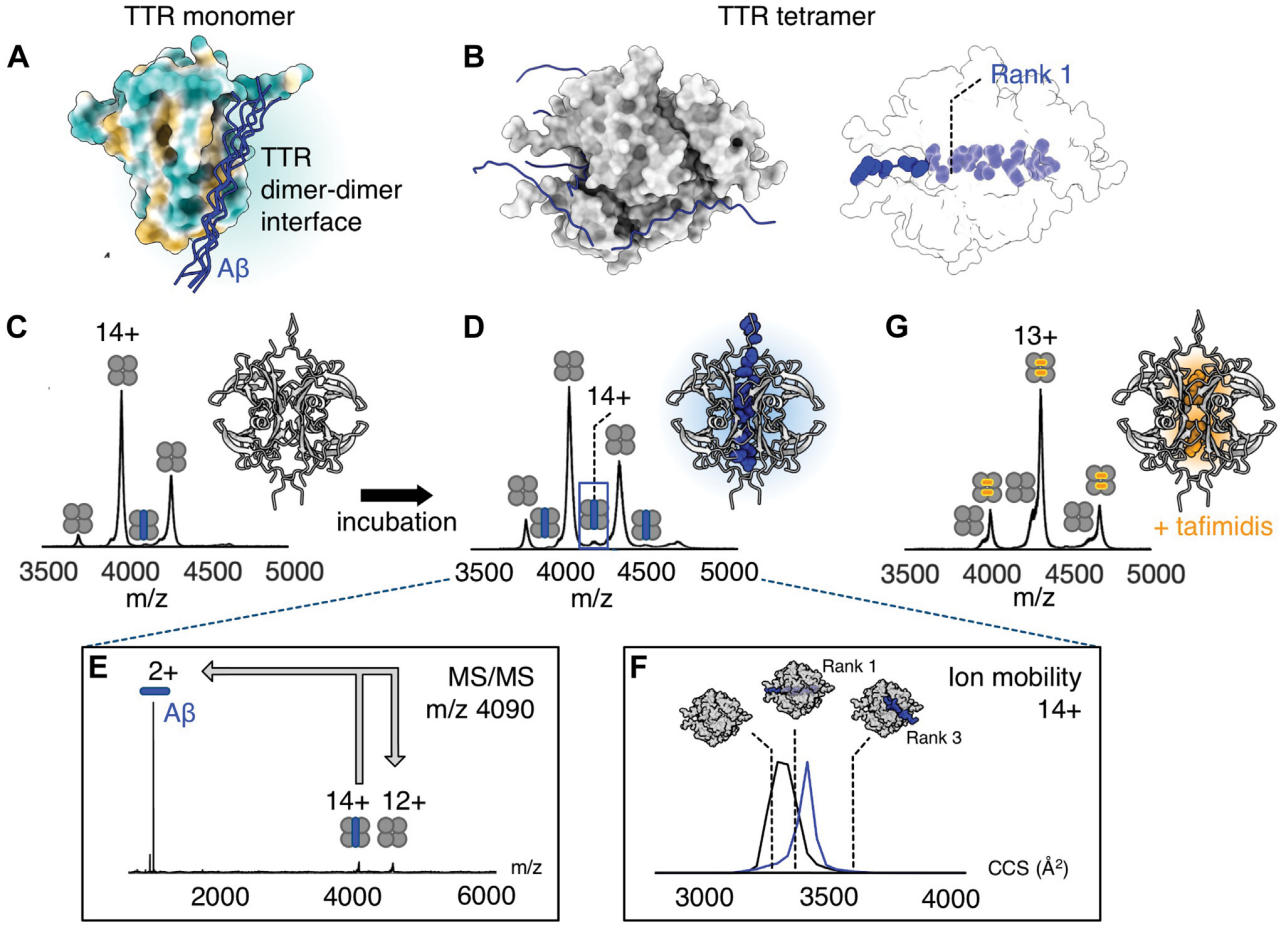
**Interactions between Aβ and TTR.***A*, AF2 predictions of the Aβ(12–28) segment interacting with the TTR monomer. The five top-scoring AF2 models are shown in a *blue cartoon* representation, and TTR is shown in a surface representation colored according to hydrophobicity. *B*, AF2 prediction of Aβ(12–28) binding to the TTR tetramer shows the positioning of the peptide in the central hydrophobic channel (rank 1), far away from the TTR tetramer (rank 2) and close to the surface of the TTR tetramer (ranks 3–5). *C*, native MS spectrum of TTR and Aβ(12–28) at a 1:1 ratio just after mixing shows a weak signal of a TTR–Aβ heterocomplex, with TTR shown as *gray circles*, whereas Aβ is illustrated as a *blue rod*. *D*, signals for TTR–Aβ heterocomplexes increase in intensity after incubation overnight at room temperature. *E*, the identity of the heterocomplex was confirmed using MS/MS. *F*, ion mobility measurements show that the CCS of the heterocomplex is closer to the AF2 rank 1 structure with Aβ bound inside the TTR tetramer, rather than the AF2 rank 3 structure with Aβ bound on the surface of TTR. The arrival time distribution of apo-TTR is shown in *gray*, that of the TTR-Aβ(12–28) complex in *blue*. *G*, further evidence for binding in the central channel is given by the minimal Aβ binding to TTR after overnight incubation, when the tetramer is stabilized by coincubation with tafamidis. Aβ, amyloid β; AF2, AlphaFold2; CCS, collision cross-section; TTR, transthyretin.

Having established that AF2 and MS accurately reflect the nonspecific nature of βLG chaperoning, we turned to TTR. It was recently reported that the antiamyloid activity of TTR is inversely correlated to the thermodynamic stability of its tetramer state (13), suggesting that binding to exposed hydrophobic surfaces on the dissociated monomer could be of importance. AF2 models of Aβ with different TTR species indicate binding of Aβ to hydrophobic patches in TTR monomers and dimers at the dimer–dimer interface (supplemental Fig. S3), in close analogy to what was observed for βLG. The central hydrophobic segment Aβ(12–28) displayed the most specific binding mode (Figs. 2*A* and S3), in agreement with modeling in a previous study (16). AF2 modeling of tetrameric TTR in complex with the monomer of this Aβ segment resulted in a top-scoring model where the Aβ(12–28) binds in the central hydrophobic channel at the TTR dimer–dimer interface, whereas the other four AF2 models positioned the peptide randomly around the TTR tetramer (Fig. 2*B*). We next performed native MS to experimentally test these predictions. Weak binding of Aβ(12–28) to TTR at a 1:4 stoichiometry was observed, with the intensity of the peaks increasing upon overnight incubation at room temperature (Fig. 2, *C* and *D*). Complex formation was confirmed using MS/MS (Fig. 2*E*). To test whether Aβ could interact with TTR monomers, we turned to the TTR_F87M,L110M_ mutant, which is a constitutive monomer in solution (34). We found that the peptide readily bound to the TTR mutant, in good agreement with previous studies (supplemental Fig. S3) (35). We next employed IM analysis of the Aβ–TTR 1:4 heterocomplex to compare the calculated CCS values for the predicted models to experimental values. The experimental CCS (3418 Å^2^) for the 14+ charge state of the 1:4 complex agreed best with the top-scoring prediction by AF2 (3374 Å^2^), where the peptide is bound in the hydrophobic pocket at the dimer–dimer interface (Fig. 2*F*). The model with Aβ bound to the surface of the TTR tetramer had a significantly larger CCS (3610 Å^2^) than that observed experimentally. The CCS values do not imply a specific architecture, but since TTR undergoes little compaction in the gas phase (36), the data support the top-scoring AF prediction. Native MS thereby confirms Aβ monomer binding to the TTR tetramer, and IM measurements enable us to accept or reject predicted complex architectures.

Binding of Aβ to the hydrophobic pocket is likely dependent on dissociation of the TTR tetramer. The influence of subunit exchange rate on complex formation was tested experimentally by adding the small organic drug molecule tafamidis, which increases the stability of the tetrameric form of TTR (37). In the presence of tafamidis, we observed a clear decrease in Aβ binding to TTR (Fig. 2*G*) after incubation. This finding suggests that the stabilized form of TTR is unable to bind Aβ peptides, where the hydrophobic patches, suggested by AF2 as favorable binding sites, are inaccessible. In summary, the AF2 predictions and IM-MS experiments support a model where dissociated TTR monomers bind monomeric Aβ through hydrophobic interactions between the TTR dimer–dimer interface and the central hydrophobic core of Aβ.

Next, we asked whether a similar mechanism could be observed for T80, which has antiamyloid activity *in vitro* and is implicated in AD. AF2 predictions of monomeric T80 and Aβ indeed suggest binding to this region (supplemental Fig. S4). Predicting the complex between dimeric T80 and Aβ yielded a sandwich-type structure in which the Aβ peptide is located between the T80 protomers. We tested this unusual model with native MS using the same approach as for βLG and TTR. Under native conditions, T80 forms mostly dimers and tetramers, but no binding of Aβ could be detected (supplemental Fig. S4). However, when T80 and Aβ were incubated at 95 °C for 30 min prior to MS analysis, we observed increased monomerization of T80 as well as minor adduct peaks corresponding in mass to a 1:1 complex (supplemental Fig. S4). We conclude that in T80 dimers, the hydrophobic patch at the dimer interface is inaccessible to interactions with monomeric Aβ. We speculate that for T80 to exert an antiamyloid activity, like βLG, Aβ would be required to encounter T80 monomers, for example, during dimer formation *via* the α-secretase cleavage.

### Pro-SP-C BRICHOS Binds Polyvaline Sequences Through β-Strand Trapping

For SP-C and CTC, although a highly specific chaperone–client pair, it is not yet clear whether selectivity is governed solely by the fact that they are synthesized as part of the same polypeptide chain, or whether CTC in addition displays sequence preferences. Recent data support that proSP-C BRICHOS binds its target polyvaline stretch in the transmembrane domain, and the mutations in the proSP-C BRICHOS domain result in incorrect folding of the polyvaline transmembrane region (38). We have previously turned to MS to establish client preferences and interaction sites in CTC, and using native MS and model peptide ligands, we found that CTC preferentially binds to hydrophobic peptides with a high β-strand propensity, with polyvaline displaying the highest relative affinity. Interestingly, peptide binding was observed with CTC monomers even when the crystal structure shows a trimer (19, 20, 21). Using HDX-MS, we furthermore found that a polyvaline ligand (KKVVVVVVVKK, termed V7), as well as a VLEM motif (residues 68–71) in the disordered region of CTC, become ordered upon binding, suggesting that V7 and the disordered region form complimentary structures (21).

**Fig. 3:**
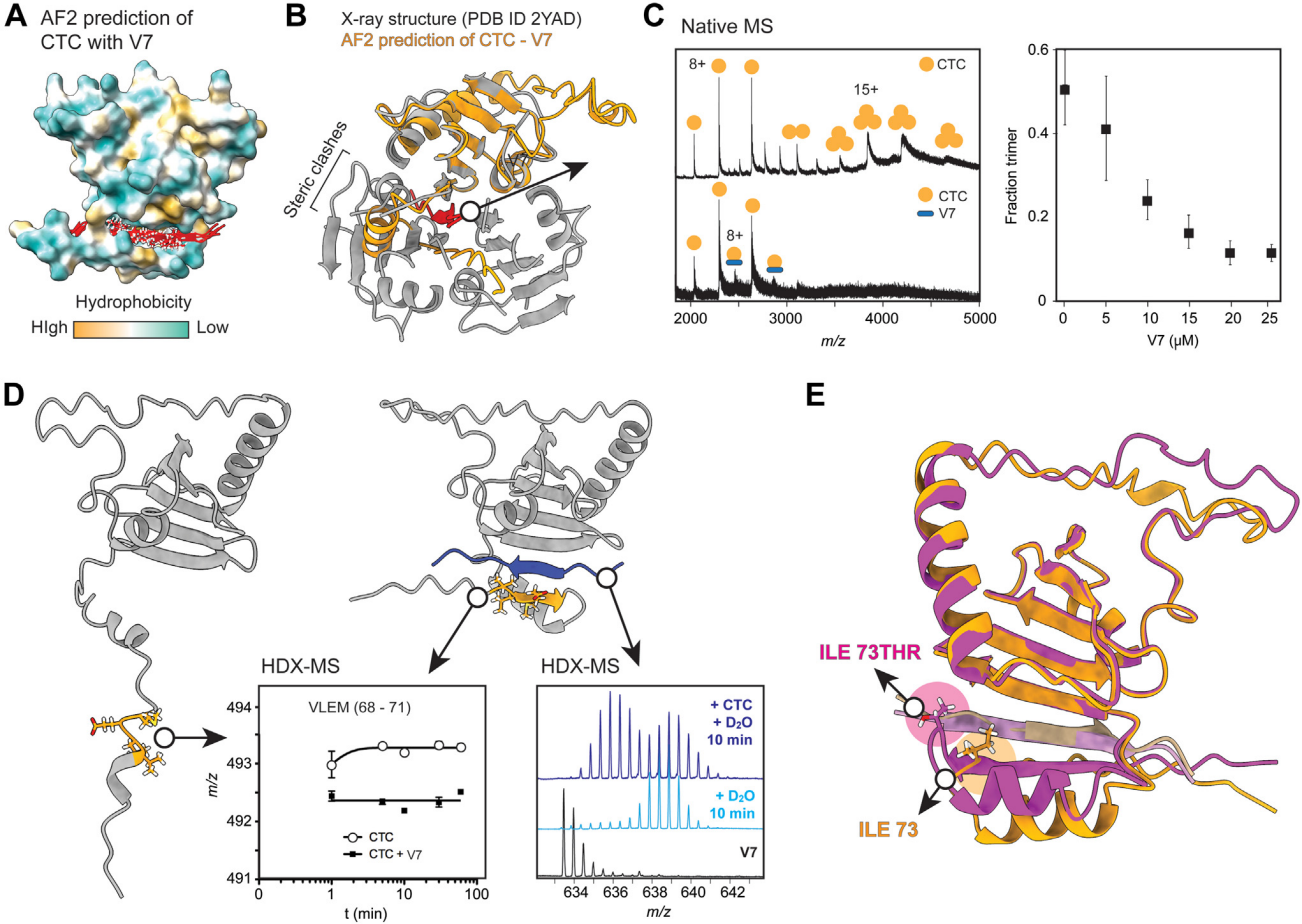
**Recognition of polyvaline by the CTC.***A*, overlays of the top five CTC–V7 complex predictions show consistent placement of the V7 ligand in a groove formed by the BRICHOS domain and the disordered region. CTC is shown as a hydrophobicity surface rendering and V7 as a *red ribbon* with valine residues as *sticks*. *B*, overlay of the proSP-C BRICHOS crystal structure (*gray*) with the AF2 model of CTC bound to V7 (*orange* and *red*, respectively) shows clashes between the disordered region of CTC and the BRICHOS trimer. *C*, native MS of CTC shows binding of V7 to monomeric CTC. Plotting the relative abundances of trimeric CTC in mass spectra recorded at increasing V7 concentrations reveals a trimer-to-monomer transition, with error bars representing the standard deviation from three independent experiments. *D*, AF2 models of ligand-free CTC (*left*) and CTC bound to V7 (*right*) suggest that V7 and the VLEM motif (residues 66–71 in proSP-C) form complementary β-strands. HDX-MS shows increased protection of both peptides from deuteration upon complex formation. *E*, overlay of AF2 predictions of wildtype CTC (*orange*) and the I73T mutant, *pink*, showing a loss of the hydrophobic contact between I73 and the polyvaline ligand as well as an upward shift of the disordered region in the mutant. I73 and T73 are shown as *sticks*. AF2, AlphaFold2; CTC, C-terminal region of proSP-C; HDX-MS, hydrogen–deuterium exchange coupled with mass spectrometry; proSP-C, proform of lung surfactant protein C.

We used AF2 to generate a model of the full-length CTC monomer bound to V7 for comparison to the crystal structure of the proSP-C BRICHOS domain (Fig. 3*A*). AF2 accurately predicted both the BRICHOS structure and the disordered loop region between the two helices. The V7 peptide was consistently predicted to bind to a groove formed by the BRICHOS domain and the disordered region that folds back on the BRICHOS domain (Fig. 3*A*). This specific structural rearrangement is in contrast to the random placement of Aβ on the surface of βLG (Fig. 1, *A* and *B*) and TTR. Alignment of the resulting complex with the trimeric BRICHOS crystal structure shows extensive steric clashes (Fig. 3*B*).

To test whether V7 binding is indeed incompatible with CTC trimerization, we recorded native MS spectra of CTC in the presence of increasing amounts of V7 (Fig. 3*C*). In agreement with previous studies (39), CTC formed trimers and monomers as well as a low amount of dimers that we attributed to in-source dissociation of trimers. The amount of CTC trimers decreased below a protein:ligand ratio of 3:1 (Figs. 3*C* and S5). V7 was found to interact with CTC monomers, although only a small fraction of CTC retained the V7 ligand in the gas phase. We have previously used native MS to determine relative affinities of CTC to model peptides and found that polyvaline exhibited the lowest gas-phase *K*_*d*_, but underwent significant in-source dissociation (20), providing a possible rationale for this observation. We conclude that V7 likely interacts both with the folded BRICHOS domain and the disordered region in CTC.

For a more detailed view of such a potential interaction, we reconsidered our previous HDX-MS data: in the absence of V7, the VLEM peptide (the central region of the disordered part) is subject to rapid deuterium incorporation, reaching full labeling within 1 min (Fig. 3*D*) (21). Notably, the VLEM peptide is the only region of CTC where we could detect an interaction with V7 by HDX-MS (supplemental Fig. S6). In the presence of V7, the VLEM peptide displays a maximum labeling of less than 50% (21). Similarly, the V7 ligand becomes nearly fully deuterated in the absence of CTC but exhibits significant protection from labeling when coincubated with CTC (Fig. 3*D*). In the AF2 model, the VLEM region forms a β-strand that runs antiparallel to the V7 peptide, which in turn binds as an extension to the central β-sheet of the BRICHOS domain. Notably, the side chains of V7 made only sparse contacts with the surrounding residues in CTC.

Together, these data indicate that binding to CTC is mostly driven by the pronounced β-sheet propensity of the ligand. To test this hypothesis, we performed AF2 and HDX-MS analysis of CTC with a helical peptide ligand (KKAAAAAAAKK, termed A7). AF2 models show no binding of A7 to any defined region of CTC with the disordered region in random orientations (supplemental Fig. S7). This result is in good agreement with previous data showing that CTC binds differently, depending on the secondary structure of the substrate peptide (40), and HDX-MS revealed no increased protection of A7 in the presence of CTC. Similarly, the VLEM motif showed complete labeling even upon coincubation with A7 (supplemental Fig. S7).

The finding that the central part of the disordered region forms a complementary β-strand pair with the polyvaline substrate has an interesting implication: one of the most prevalent genetic defects linked to interstitial lung disease is a missense mutation in proSP-C leading to the replacement of isoleucine 73 with a threonine directly adjacent to the VLEM motif (I73T) (41, 42). Based on the excellent agreement between the AF2 models and the MS data, we decided to delineate the effect of the I73T mutation on ligand binding using AF2 (Fig. 3*E*). The model shows that the mutation leads to a loss of hydrophobic interactions between isoleucine 73 and the C-terminal valine residue of V7. Instead, T73 appears to hydrogen bond with the ligand backbone, pulling the rest of the disordered region upward. We conclude that the mutation interferes with the ability of CTC to keep its ligand in an extended conformation, providing a rationale for its effect on pathogenic SP-C misfolding (42).

A high propensity to fold into β-sheet structures is a common property of amyloidogenic peptides/proteins, and the mechanism observed here for CTC/V7 appears to be a general mechanism for antiamyloid activity in chaperones. A structurally similar example is DNAJB6, a Hsp40-type chaperone that specifically binds amyloidogenic peptides including polyQ peptides and Aβ (43, 44, 45). All Hsp40 chaperones consist of a highly conserved N-terminal J-domain, which recruits Hsp70, and a client binding C-terminal domain, which is specific for the client protein of the chaperone. The C-terminal domain in DNAJB6 forms a single β-sheet that could bind clients with β-sheet propensity. AF2 modeling of Aβ and the C-terminal domain of DNAJB6 does indeed predict highly specific binding of Aβ in a β-hairpin conformation to the edge strand of the chaperone β-sheet (supplemental Fig. S7). This binding site is not a hydrophobic interface, as the edge strand is rich in polar serine and threonine residues known to be of importance for the antiamyloid activity of the chaperone (46, 47). Such specific binding is therefore driven by complementary conformations rather than hydrophobic interactions.

## Conclusions

In this study, we have combined AF2 structure prediction with native MS, IM-MS, and HDX-MS to compare nonspecific interactions with specific chaperone–peptide interactions. AF2 and IM-MS show that the nonspecific chaperone activity of βLG and TTR toward Aβ is due to hydrophobic interactions between the peptide and the proteins. Access to hydrophobic surfaces can be subject to regulation *via* conformational dynamics. Consequently, very stable assemblies such as T80 and tafamidis-stabilized TTR appear to be poorer binders of amyloid peptides. On the other hand, the specific interaction between the polyvaline region of SP-C and its C-terminal BRICHOS domain is driven by the strong β-strand propensity of the ligand, which is trapped by a complementary β-strand of the disordered region of proSP-C, as shown here by AF2, native MS, and HDX-MS. This binding mechanism is similar to those suggested for other dedicated antiamyloid chaperones, such as the DNAJB6 chaperone. In summary, the combination of machine learning and MS-based structural proteomics now reveals the details of previously inaccessible protein interactions. We anticipate that this methodology, in the future, will allow successful modeling of further relevant protein complexes, such as peptide–drug binding, and hormone–receptor interactions.

## Data Availability

The raw IM-MS and HDX-MS data in PULSAR and .txt formats, respectively, are available from the DRYAD repository, https://doi.org/10.5061/dryad.r2280gbgj.

## Supplemental data

This article contains supplemental data.

## Conflict of interest

The authors declare no competing interests.
