# Supplementary material for: Mass Spectrometry and Machine Learning Reveal Determinants of Client Recognition by Antiamyloid Chaperones
Source: Mol Cell Proteomics. 2022 Sep 15;21(10):100413. doi: 10.1016/j.mcpro.2022.100413 (PMC9563204; doi:10.1016/j.mcpro.2022.100413)
Supplement: Supplemental data [file mmc1.pdf]

Supplementary Information to:

Mass spectrometry and machine learning reveal determinants of client recognition by anti-amyloid chaperones

Nicklas Österlund, Thibault Vosselman, Axel Leppert, Astrid Gräslund, Hans Jörnvall, Leopold L. Ilag, Erik G. Marklund, Arne Elofsson, Jan Johansson, Cagla Sahin, and Michael Landreh

Table S1

Figures S1-S7

Table S1. Protein and peptide concentrations used for native MS.

Protein – ligand pair	Protein concentration (μM)	Ligand concentration (μM)
Aβ + βLG	30	30
$A\beta_{12\text{-}28}$ + TTR	20	20
Aβ + $TTR_{F87M,\ L110M}$	20	20
Aβ + T80	27	15
V7 + CTC	20	0 - 20

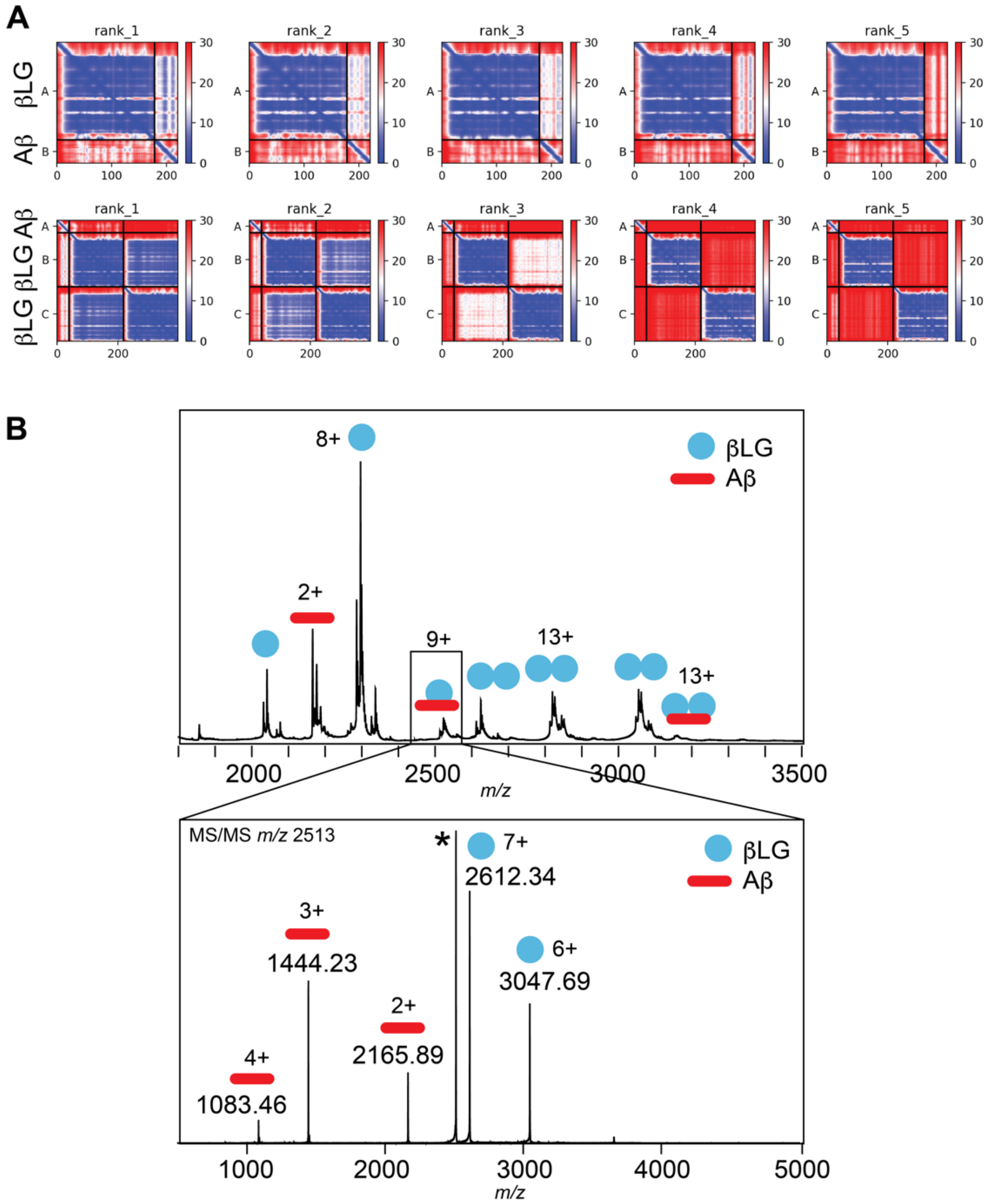

Figure S1. (A) PAE plots for the top five models for the complex between Aβ and βLG monomers (top) and dimers (bottom) show only diffuse contacts with high positional error, suggesting non-specific interactions. The lowest-scoring models show disruption of the dimer interface in the presence of Aβ. (B) MS/MS of the peak at *m/z* 2513 confirms the presence of both Aβ and βLG. The precursor ion is marked with an asterisk.

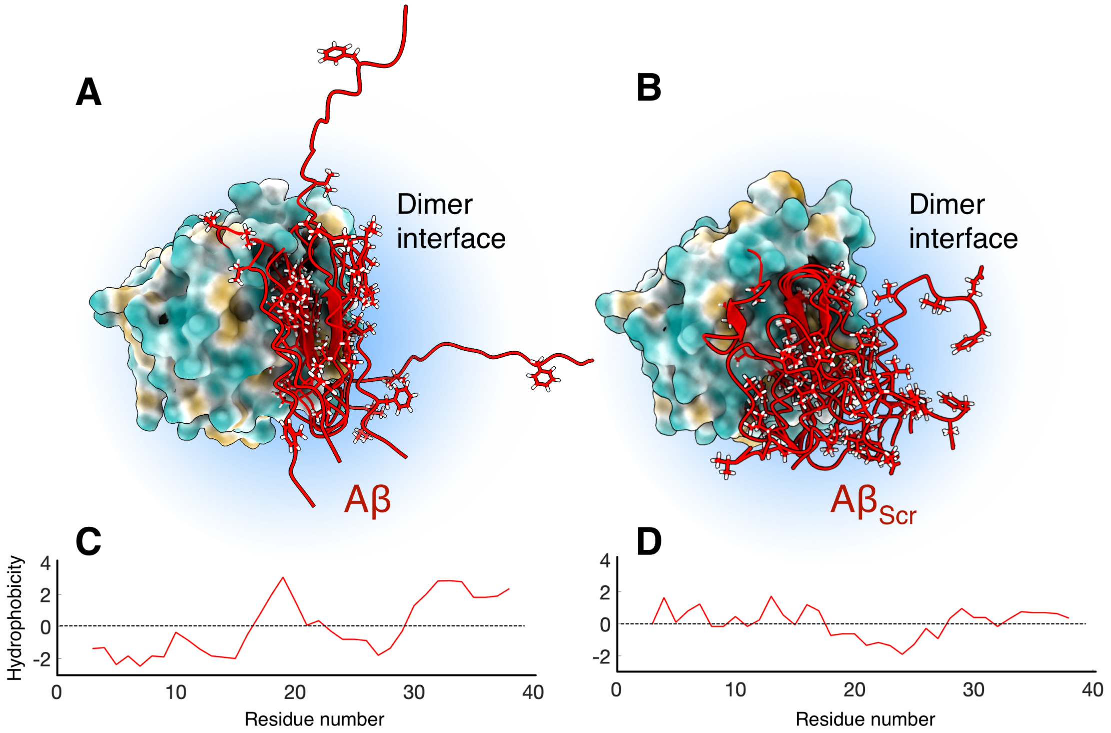

Figure S2. (A) AF2 predictions of complexes between Aβ and βLG show binding between hydrophobic parts of Aβ and hydrophobic parts βLG at the dimer interface. Aβ is shown in red cartoon representation with hydrophobic residues in stick representation, βLG is shown in surface representation colored according to hydrophobicity. (B) AF2 prediction of complexes between scrambled Aβ and βLG show similar binding between hydrophobic parts of Aβ and hydrophobic parts βLG, illustrating that the interaction between βLG and Aβ is not sequence specific. (C) and (D) show sequence hydrophobicity of Aβ and scrambled Aβ and illustrate how the hydrophobicity is more evenly distributed over the peptide sequence in scrambled Aβ compared to in Aβ where hydrophobic residues are clustered in two distinct segments. Amino acid hydrophobicity is scored according to the Kyte & Doolittle scale.

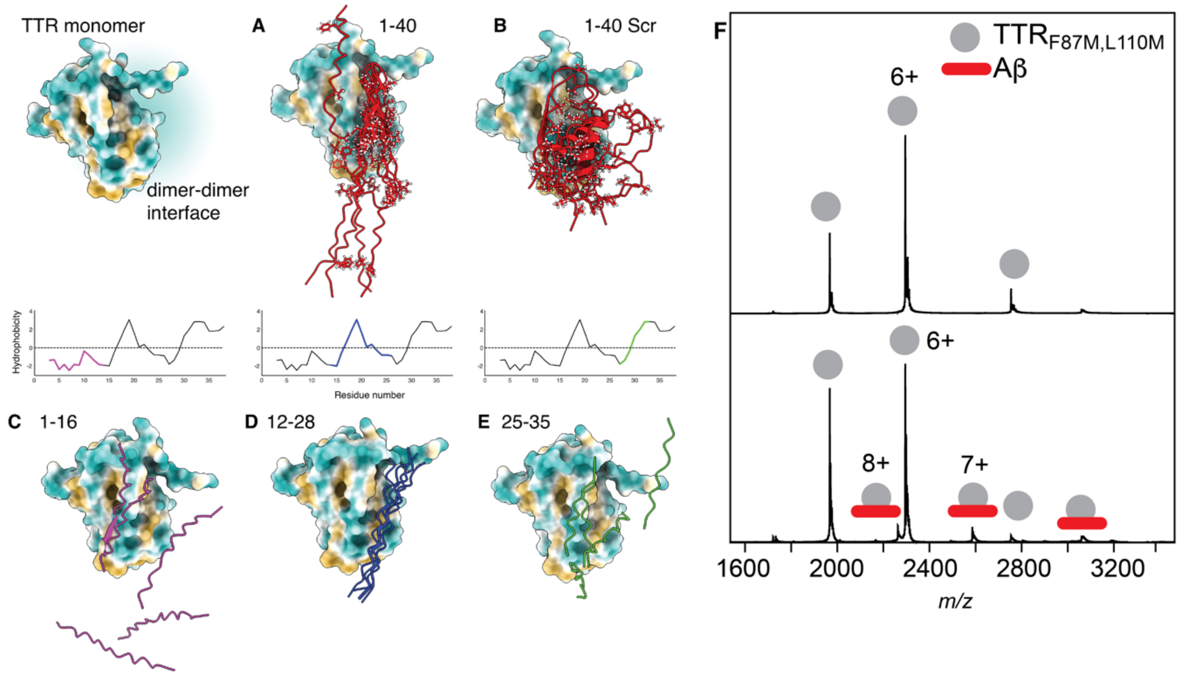

Figure S3. AF2 predictions of complexes between monomeric TTR and (A) Aβ and (B) scrambled Aβ show very similar binding modes as predicted for binding to βLG (Figure S2). Hydrophobic residues in the peptide interact with the hydrophobic dimer-dimer interface of TTR. (C-E) Sequence hydrophobicity computed using the Kyte & Doolittle scale, and the corresponding AF2 predictions for binding to TTR are shown for three Aβ segments: (C) the hydrophilic N-terminal Aβ(1-16) segment (D) the hydrophobic central Aβ(12-28) segment and (E) the C-terminal Aβ(25-35) segment. The 12-28 segment shows the best-defined binding to the TTR monomer. (F) Mass spectra of the monomeric $TTR_{F87M,L110M}$ in the absence (top) or presence (bottom) of Aβ shows the formation of a 1:1 complex.

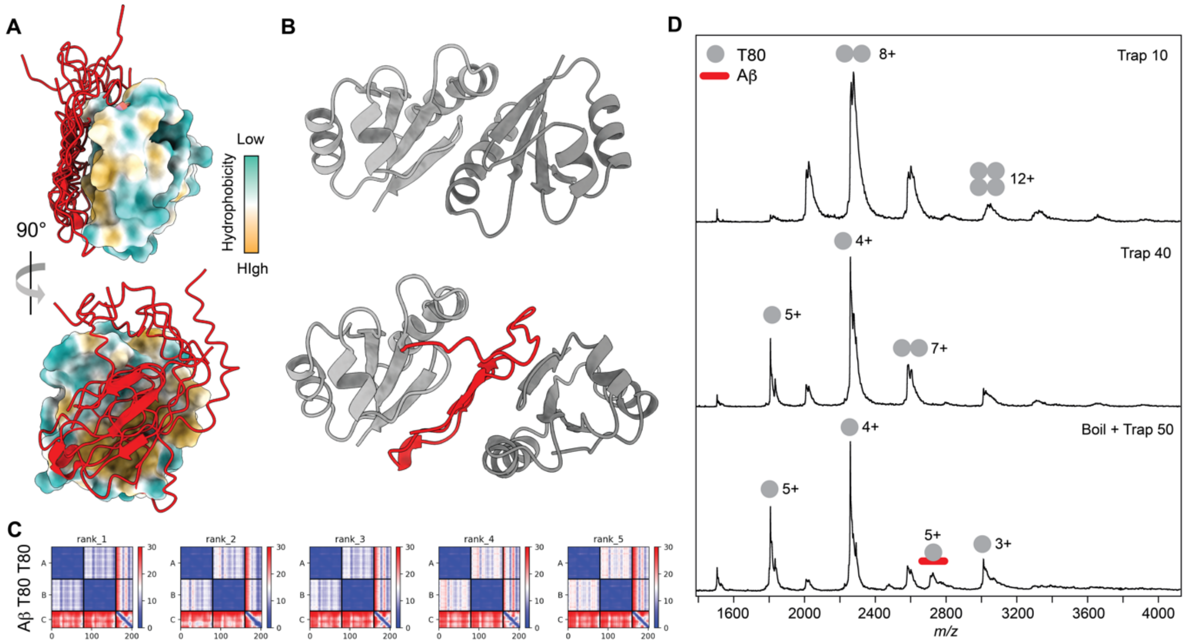

Figure S4. (A) AF2 models of Aβ bound to T80 monomers show predominant location of Aβ on the hydrophobic patch exposed by cleavage of the C-terminal helix of thioredoxin. (B) AF2 models of dimeric T80 (top) and Aβ bound to two T80 molecules (bottom) propose a “sandwich”-type interaction, where the peptide (red) interacts with hydrophobic regions normally buried at the dimer interface. (C) PAE plots for the top five models of dimeric T80 with Aβ show consistent placement of the peptide between the dimer subunits. (D) Native MS in the presence of Aβ show no interaction under gentle MS conditions (top). Increased collisional activation results in peak sharpening but reveals no Aβ adducts (middle). Incubation of T80 with Aβ at 95°C prior to MS analysis results in T80 monomerization as well as the formation of a minor amount of 1:1 complexes between Aβ and T80 (bottom).

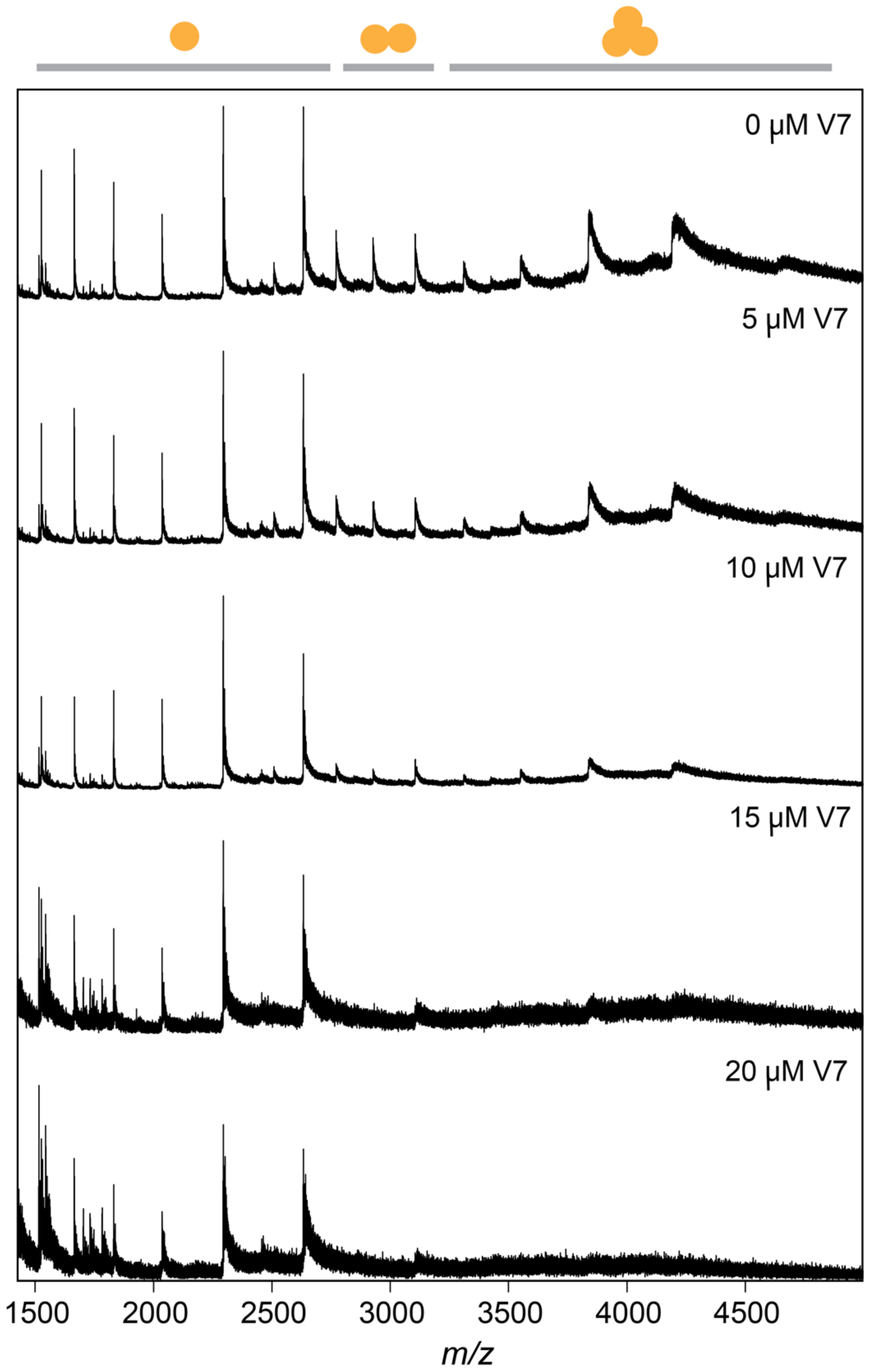

Figure S5. Native mass spectra of CTC in the presence of increasing amounts of V7 show gradual reduction in the amount of trimers. We also detect a minor dimer population, which likely stem from in-source dissociation of the trimers.

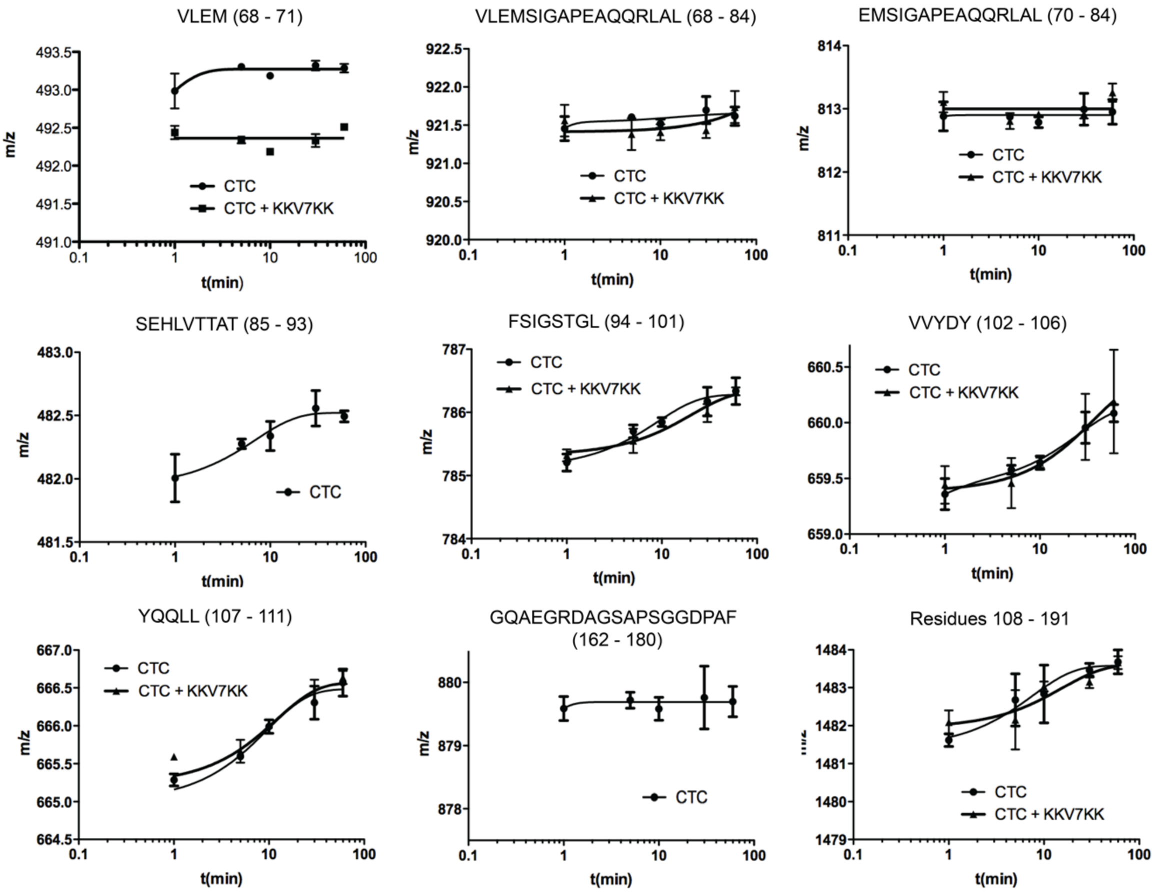

Figure S6. Deuterium uptake for all peptic peptides of CTC with and without V7 show that only the VLEM motif is affected by V7 binding. The fragments covering residues 162-180 and 85-93 could not be detected in the presence of V7. Data were recorded and analysed as described [21]. Error bars indicate the standard deviation of three independent repeats.

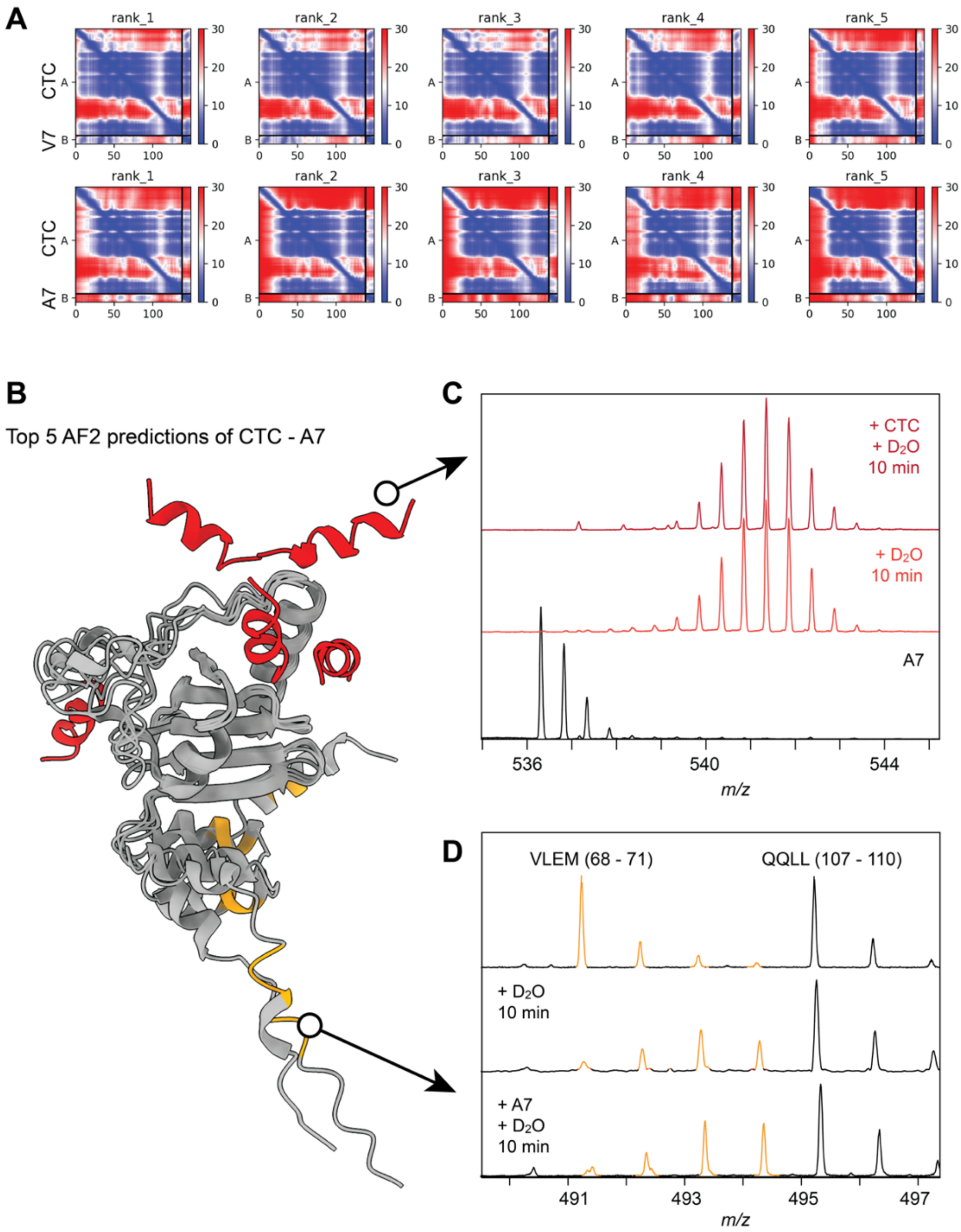

Figure S7. (A) PAE plots of five models of CTC and V7 (top) or CTC and A7 (bottom) show pronounced interactions between V7 and the N-terminal, disordered region of CTC. No such interactions are observed for complexes with A7. (B) Overlay of the top five complexes predicted for A7 with CTC show random placement of the A7 peptide (red), and no interactions with the VLEM motif in the disordered region (orange). (C) HDX-MS data show no difference in deuterium uptake for the A7 ligand in the absence or presence of CTC. (D) The VLEM peptide displays nearly full deuteration with and without A7 present.

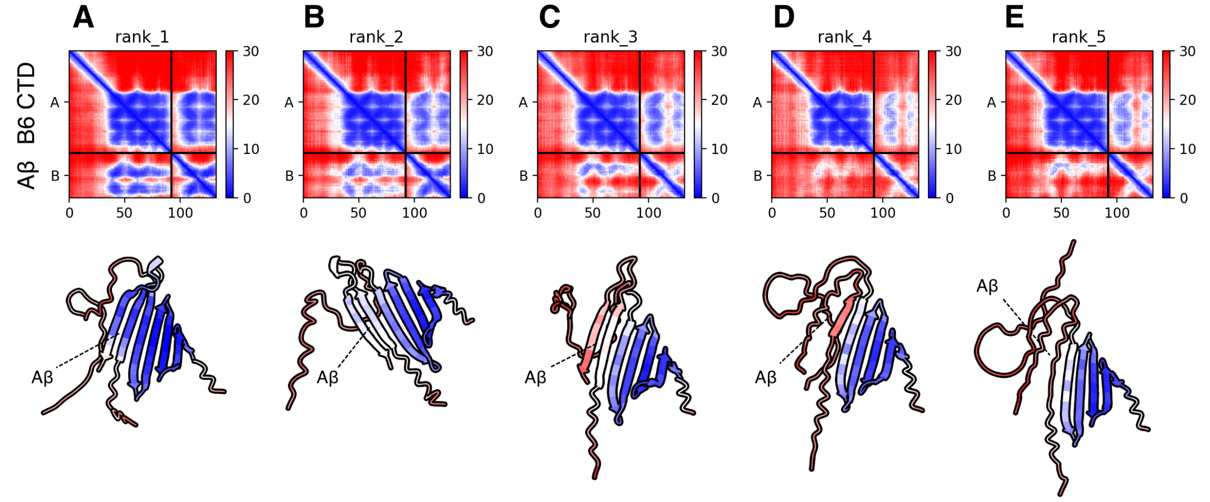

Figure S7. AF2 predicts a specific structural rearrangement where Aβ binds in a β-hairpin conformation to the β-sheet structured client-binding C-terminal domain of the DNAJB6 chaperone. The Aβ-B6 CTD complexes are colored according to PAE score.
